# Theoretical and Experimental Studies of *N*,*N*-Dimethyl-*N′*-Picryl-4,4′-Stilbenediamine

**DOI:** 10.1007/s10895-014-1425-9

**Published:** 2014-08-09

**Authors:** Vladislav Papper, Yuanyuan Wu, Vladimir Kharlanov, Ayrine Sukharaharja, Terry W. J. Steele, Robert S. Marks

**Affiliations:** 1 School of Materials Science and Engineering, Nanyang Technological University, 50 Nanyang Avenue, Singapore, 639798 Singapore; 2 Institute of Chemistry, Humboldt University of Berlin, Berlin, Germany; 3 Department of Biotechnology Engineering, National Institute for Biotechnology in the Negev, Ilse Kats Institute for Nanoscale Science and Technology, Ben Gurion University of the Negev, Beer Sheva, Israel

**Keywords:** Intramolecular charge transfer, Fluorescence

## Abstract

*N,N*-dimethyl-*N′*-picryl-4,4′-stilbenediamine (DMPSDA) was prepared, purified and crystallised in a form of black lustrous crystals, and its absorption and fluorescence spectra were recorded in cyclohexane, acetonitrile and dimethyl sulfoxide. Non-emissive intramolecular charge transfer state (ICT) was clearly observed in this molecule in all three solvents. Theoretical calculations demonstrating a betaine electronic structure of the trinitrophenyl group in the ground state of the molecule and a charge transfer nature of the long wavelength transition S_0_ → S_1_ supported the experimental observations of the ICT formation in the molecule.

## Introduction

We have previously discussed various time-scale processes occurring with 4,4′-disubstituted stilbenes after their irradiation [1–3]. We found that different 4,4′-disubstituted stilbenes exhibit different sensitivity to intramolecular donor-acceptor effects of substituents and medium polarity. All the investigated 4,4′-disubstituted stilbenes were divided into three groups according to their intramolecular stabilization of the excited ^1^ t* state. Those having strong donor dimethylamino (DMA) group at one phenyl ring and strong acceptor substituent at another phenyl ring constituted the third group called “push-pull stilbenes”. They were characterised by large charge delocalisation between donor and acceptor aromatic moieties, large dipole moment, low polarity ^1^p* state with preferential stabilisation of ^1^ t* compared to less polar ^1^p* state, very high activation barrier for the ^1^ t* → ^1^p* twisted transition, and net increase of the fluorescence quantum yield and fluorescence life-time with increase of solvent polarity. All these observations indicated completely different non-radiative relaxation pathway than the ^1^ t* → ^1^p* twisted transition observed in most stilbene compounds, which are not classified as “push-pull stilbenes” [1–3].

Actually, the third group has been formed by three investigated “push-pull stilbenes”*,* namely 4-dimethylamino-4′-cyanostilbene (DACS), 4-dimethylamino-4′-carbomethoxystilbene (DACMS) and 4-dimethylamino-4′-nitrostilbene (DANS). These three stilbenes exhibited very large Stock shifts, compared to the first and second groups [1–3]. In that case, the highly polarized excited state, which creates a huge dipole moment, is stabilised extensively by polar interactions (Stock shift value ∆E was found to be approximately 22–23 kcal/mol). Experimental observations of high fluorescence quantum yields and long fluorescence lifetimes in strongly polar solvents indicated a strongly preferred pathway toward the intramolecular charge transfer (ICT) formation in the competing photochemical processes starting from ^1^ t*. Assuming that this point holds in less polar solvents as well, the reduced fluorescence quantum yield and increased non-radiative decay rate is probably due to the insufficient stabilization of the ICT in these solvents. This can easily be understood by assuming that ^1^p* is less polar than ICT and ^1^ t* states, and thus increasing solvent polarity preferentially lowers a highly polar ICT state with respect to ^1^p*.

Although three aforementioned “push-pull” stilbenes were categorised into one group, they do not necessarily conform to the same relaxation mechanism. In fact, intramolecular charge transfer in these molecules may be observed as an emissive or non-emissive process dependent on the 4,4′-substitution pattern. In case of DANS, we assumed a non-emissive ICT state, which was attributed to a specific interaction of the nitro-group, which quenches the charge-transfer state emission [4, 5]. Actually, non-emissive ICT states were observed in polar solvents when the acceptor group is very powerful and has a low-lying anti-bonding orbital [4]. To generalise this observation, we prepared and studied a new “push-pull” stilbene having a relatively strong acceptor substituent, namely trinitrophenyl (TNP) group. This molecule has been disclosed only once in literature and only its UV absorption spectrum has been previously reported [6].

Herein, we report the synthetic preparation and experimental studies of this molecule, as well as theoretical calculations aimed at elucidating the absolute geometry of the most stable conformer, which might be responsible for the experimentally observed charge-transfer interactions in the ground state of the molecule.

## Experimental

### Reactants and Solvents

Reactants were either commercially available or freshly prepared as detailed in the *Synthesis* section. Commercially available organic solvents, namely cyclohexane (Sigma-Aldrich ACS spectrophotometric grade, ≥99 %), dimethyl sulfoxide (Sigma-Aldrich ACS spectrophotometric grade, ≥99 %) and acetonitrile (Sigma-Aldrich ACS spectrophotometric grade, ≥99 %), used for spectroscopy did not require any additional purification.

### Apparatus and Methods

Semi-preparative flash chromatography used for purification of the compounds was performed with the Büchi Sepacore® flash chromatography system using 25 g silica gel cartridges (particle size 40–63 μm) and dichloromethane as an eluting solvent. ^1^H NMR spectra were run on 10 % (w/v) sample solutions in CDCl_3_ with (CH_3_) _4_Si as an internal standard at room temperature using a 400 MHz Bruker Fourier transform spectrometer, equipped with a DMX AVANCE I system. UV absorption spectra were measured using an Agilent Cary 300 spectrophotometer, and the steady-state fluorescence spectra were recorded with Horiba Jobin Yvon FluoroLog®-3 modular spectrofluorometer and Agilent Cary Eclipse fluorescence spectrophotometer. The fluorescence excitation and emission spectra were corrected for instrumental sensitivity at different excitation and emission slits using the instrument internal excitation-emission matrix (EEM) correction [7]. A solution of quinine bisulphate in 0.1 N H_2_SO_4_ (Ф_f_ = 0.52) was taken as fluorescence standard for the determination of fluorescence quantum yields [8]. Constant-illumination fluorescence intensity decay curves at the photostationary steady-state equilibrium between the *trans*- and *cis*-isomers of DMPSDA were recorded with Shimadzu RF-5,301 spectrofluorometer equipped with the 150 W Xenon lamp as a radiation light source. The fluorescence decay at the photostationary steady-state equilibrium was monitored at the emission maximum of DMPSDA after excitation at the excitation maximum using typically 5-nm slit width for excitation and 5-nm slit width for emission. Analysis of the experimental data was performed using Origin® Pro 9.0 for Windows.

Fluorescence intensity decay curves were analysed with a polynomial fit for calculation of the *trans-cis* isomerization rate constants using a self-written routine within the Origin® Pro 9.0 for analysis of the first-order photochemical reaction rates.

### Synthesis of N,N-Dimethyl-N′-Picryl-4,4′-Stilbenediamine

The following reactants were prepared by slight modification of our procedure that we published in [9]: *trans*-4-dimethylamino-4′-nitrostilbene and *trans*-4-dimethylamino-4′-aminostilbene.

1. Trans-4-Dimethylamino-4′-nitrostilbene

An equimolar mixture of 4-nitrophenylacetic acid (Sigma-Aldrich N20204) (9.1 g) and 4-(dimethylamino) benzaldehyde (Sigma-Aldrich 156,477) (7.5 g) in 3 ml of piperidine was heated for 24 h under reflux in an oil bath maintained at 140°. The resulting dark-red crude solid was recrystallized from 100 ml of chlorobenzene. Upon cooling, the product precipitated as lustrous red flakes, then was collected by vacuum filtration on a Buchner funnel, thoroughly washed with petroleum ether (60–80°) to remove the adherent piperidine and dried in a vacuum oven at 60° for about 2 h. Yield of the lustrous red flakes – 7.4 g (53 %). Chemical purity and structural identity of the product was confirmed by ^1^H NMR.

(2) Trans-4-Dimethylamino-4′-aminostilbene

Solution of 120 g of potassium hydroxide pellets dissolved in 200 ml of ultrapure water was prepared in 500 ml Erlenmeyer flask and placed in a fridge for storing. This solution is used for decomposition of the prepared stannous complex salt of the product. 15 g of stannous chloride dihydrate was dissolved in 150 ml of concentrated hydrochloric acid at room temperature with stirring. 4 g of fresh *trans*-4-dimethylamino-4′-nitrostilbene prepared in the previous step was introduced into the stirred solution of SnCl_2_. The resulted suspension was refluxed with strong stirring for about 4–5 h until all red flakes and orange viscous particles of the intermediate stannous complex salt formed during the reaction have been completely dissolved, and the yellow reaction mixture has become transparent. The reaction mixture was stirred for additional half an hour and allowed to cool to the point of turbidity. Then it was poured carefully into a cold stirred solution of potassium hydroxide taken from the fridge and placed into an ice bath, in order to decompose the complex stannous salt and to release the product. Caution: the neutralization reaction is highly exothermic and vigour! Well-protective glasses, gloves and clothes should be worn during this step, and all precautions for minimising the reaction mixture splashing should be taken. The crude yellow product was collected by vacuum filtration, thoroughly washed with 10 % sodium bicarbonate solution, ultrapure water and then with petroleum ether (60–80°). The crude yellow product was dissolved in 10 ml of dichloromethane, and subjected to the flash chromatography on the regular silica gel (particle size 40–63 μm). The fractions containing *trans*-isomer (′_abs_ = 360 nm) were collected and evaporated to yield the yellow amorphous powder (2.82 g, 77 % reaction yield). Chemical purity and structural identity of the product was confirmed by ^1^H NMR.

(3) N,N-dimethyl-N′-picryl-4,4′-stilbenediamine

An equimolar mixture of 0.48 g (2 mmol) of *trans*-4-dimethylamino-4′-aminostilbene, prepared in a previous step, and 0.50 g (2 mmol) of 2-chloro-1,3,5-trinitrobenzene (Sigma-Aldrich 79,874) in 5 ml absolute ethanol, which yielded initially a clear solution, was left overnight with stirring The resulted black crystalline precipitate was collected by vacuum filtration on a Buchner funnel, washed with 10 % sodium bicarbonate solution, ultrapure water and then with petroleum ether (60–80°). The crude product was recrystallized from chlorobenzene, and then dried in a vacuum oven at 60° for about 2 h. Yield of the black crystalline product – 0.76 g (85 %). ^1^H NMR: δ 3.00 (Me_2_N, s, 6H); δ 6.88, δ 6.93 (CH = CH AB, d, vinyl 2H); δ 6.72, δ 7.89 (4-Me_2_N-Ar AA′XX′, dd, 4H); δ 7.33, δ 7.83 (4′-picrylamine-Ar AA′XX′, dd, 4H); δ 9.24 (picryl ring protons, s, 2H); δ 3.71 (NH).

## Results and Discussion

### Experimental Studies

**Fig. 1:**
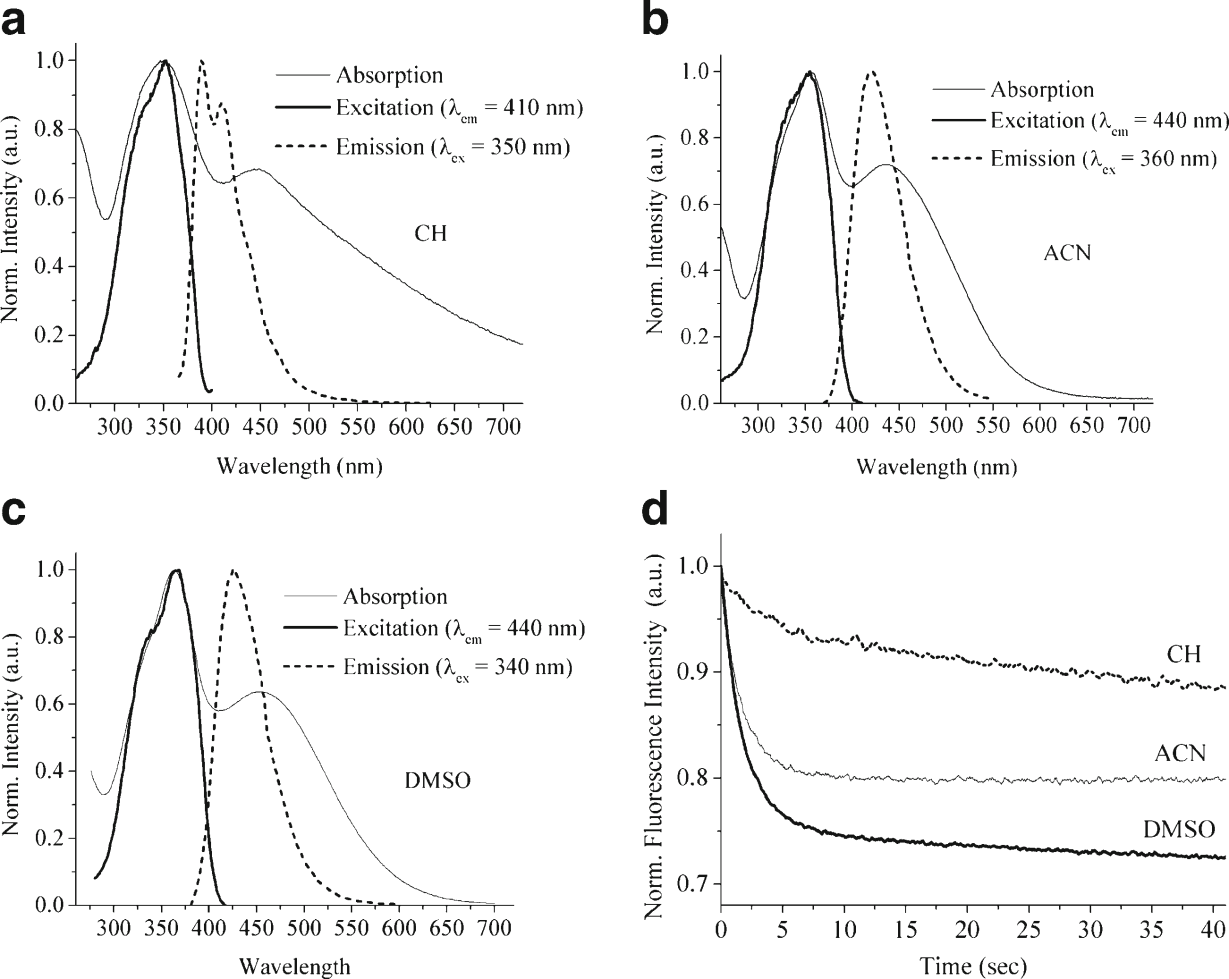
Normalised absorption, excitation and emission spectra of *trans*-DMPSDA in different solvents: **a** CH, **b** ACN and **c** DMSO, and **d** the photostationary fluorescence decay curves under constant-illumination conditions in these three solvents

**Fig. 2:**
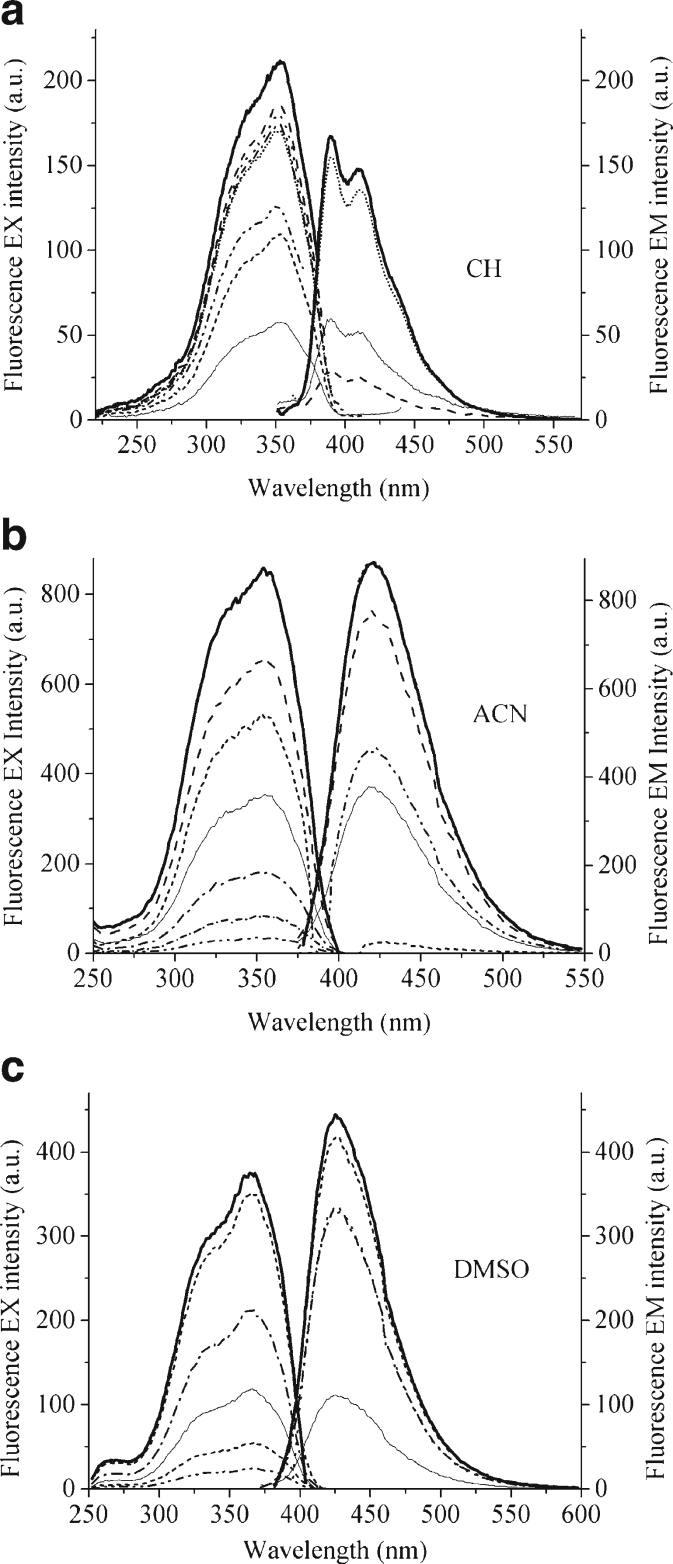
Fluorescence excitation and fluorescence emission spectra of *trans*-DMPSDA in three solvents at the different excitation and emission wavelengths: **a** CH, emission range 380–500 nm; excitation 275–350 nm; solid bold line – λ_em_ = 390 nm, λ_ex_ = 320 nm; **b** ACN, emission range 400–520 nm; excitation 300–400 nm; solid bold line – λ_em_ = 420 nm, λ_ex_ = 360 nm; and **c** DMSO, emission range 420–520 nm; excitation 300–400 nm; solid bold line – λ_em_ = 420 nm, λ_ex_ = 360 nm

We succeeded to synthesize and recrystallize a stable *trans*-isomer of DMPSDA. Figure 1 shows the absorption, excitation and emission spectra of *trans*-DMPSDA in different solvents (CH, ACN and DMSO), as well as the photostationary fluorescence decay curves under constant-illumination conditions. Figure 2 demonstrates the fluorescence excitation and emission spectra of *trans*-DMPSDA in three solvents. Table 1 brings together all the spectroscopic properties of the molecule in these solvents at room temperature including absorption, fluorescence excitation and emission maxima (λ_abs_, λ_ex_ and λ_em_), fluorescence quantum yields (Φ_f_) and apparent fluorescence decay rate constants (k_app_), for the investigated compound in three solvents at room temperature. There is a clear non-emissive ICT band observed at the longer wavelength in the absorption spectra of the molecule in all three solvents. Theoretical calculations, which will be presented in the next section, show the possible electronic structure of *trans*-DMPSDA responsible for this non-emissive ICT state.

**Table 1:** Absorption, fluorescence excitation and emission maximum wavelengths (λ_abs_, λ_ex_ and λ_em_), fluorescence quantum yields (Φ_f_) and apparent fluorescence decay rate constants (k_app_) for *N,N*-dimethyl-*N′*-picryl-4,4′-stilbenediamine in acetonitrile (ACN), dimethyl sulfoxide (DMSO) and cyclohexane (CH) at room temperature

Solvent	λ_abs_ (nm)	λ _ex_ (nm)	λ _em_ (nm)	Φ_f_	k_app_ (s^−1^)
ACN	360	360	420	0.009	0.643
DMSO	365	365	425	0.013	0.614
CH	350	350	390/410	0.001	−

The photostationary fluorescence decay of *trans*-DMPSDA is observed only in relatively polar solvents (ACN and DMSO) and originates from the non-radiative ^1^ t* → ^1^p* twisted transition, which is responsible for *trans-cis* isomerisation of the molecule and acts as a quenching funnel on fluorescence emission. CH is a non-polar solvent, and vibrational relaxation of the Franck-Condon state plays the primary role in its stabilisation of the excited state, thereby considerably slowing the ^1^ t* → ^1^p* transition [1, 2].

The measured fluorescence quantum yield values in all three solvents, shown in Table 1, are relatively low compared to the parent *trans*-4-dimethylamino-4′-aminostilbene [1–3]. We assume that part of the additional non-radiative losses are caused by the electronic energy transfer onto the TNP fragment, which intrinsically shows negligible fluorescence, due to the presence of an efficient conical intersection of the S_1_ state with the ground state S_0_ [10].

### Theoretical Calculations

Theoretical calculations were conducted with the following software suites: Gaussian 03 [11], Gaussian 09 [12] and Turbomole 6.1 [13]. The ground state studies were carried out by the density functional theory (DFT) using the Becke’s Three Parameter Hybrid Method and the correlation functional of Lee, Yang, and Parr (LYP) with the correlation potentials VWM (III) (calculated in Gaussian 03 or Gaussian 09) and VWM (V) (calculated in Turbomole 6.1). The optimized structures were controlled by frequency calculations with the vibration analysis using Hessian matrix. The excited states were calculated by the Time Dependent B3LYP hybrid method for the ground state optimized structures.

**Fig. 3:**
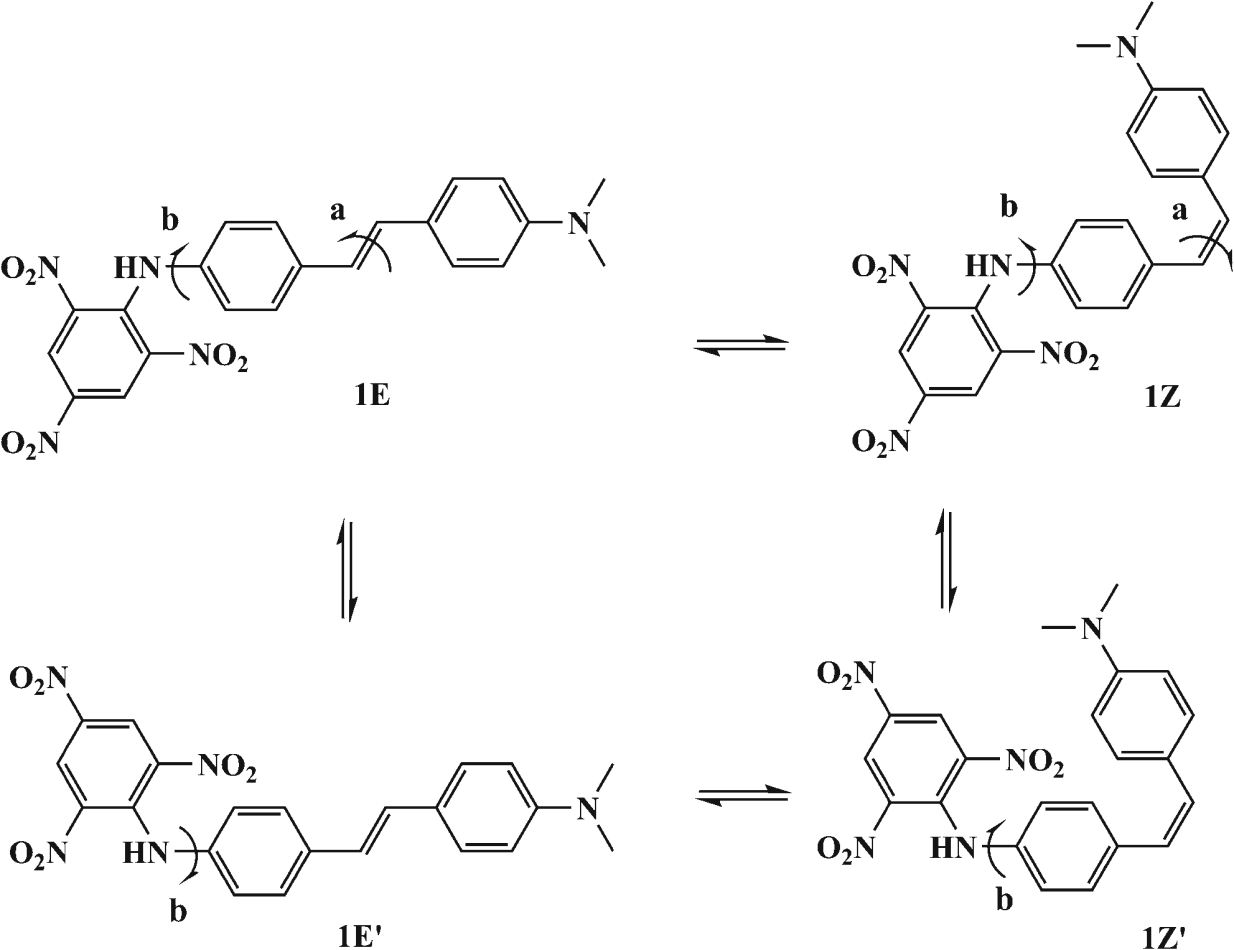
Stereoisomers 1E, 1E′, 1Z, 1Z′ of DMPSDA studied with the ab initio calculations

**Fig. 4:**
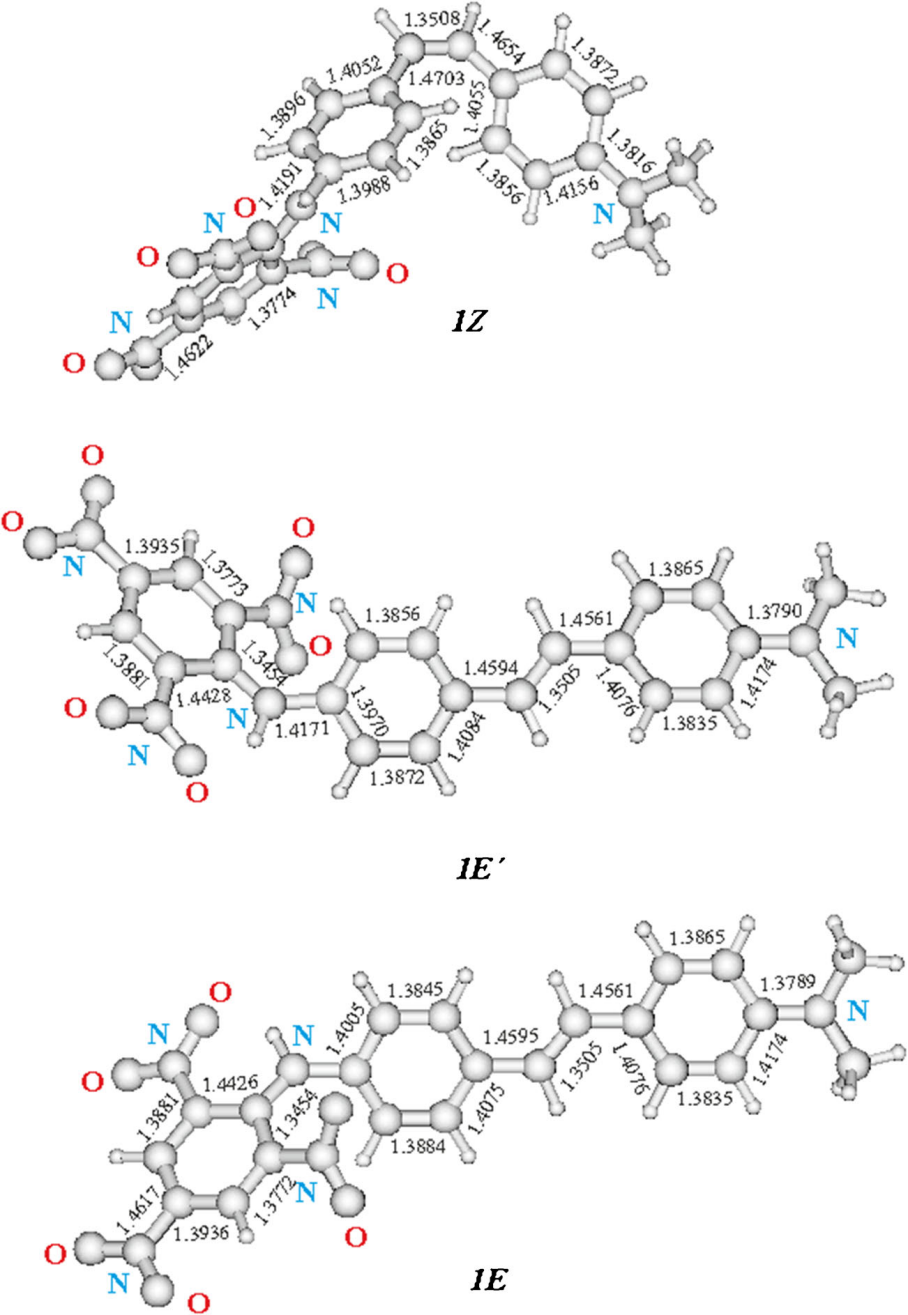
B3LYP optimized 3D structures of 1E, 1E′ and 1Z with the bond lengths

Figure 3 shows the possible isomers 1E, 1E′, 1Z, 1Z′ of DMPSDA studied with the ab initio calculations. The B3LYP optimized structures with the bond lengths are shown in Fig. 4. The calculated properties of the isomers are given in Tables 2 and 3. The obtained results clearly demonstrate that *trans*-isomer 1E is the most electronically stable isomer. However, the energy difference (∆E = 0.024 kcal/mol) to another *trans*-isomer 1E′ is very small. On the other hand, *cis*-isomer 1Z having ∆E = 5.04 kcal/mol is relatively unstable. The frequency calculations showed that all optimized isomeric structures are equilibrium ones. The vibration frequencies are positive and the number of negative Hessian is zero.

**Table 2:** Absolute energy E_h_, relative energy ∆E, dipole moment μ_g_ and number of negative Hessian of the equilibrium structures for isomers 1E, 1Z and 1E′calculated by B3LYP/6–31 + G (3df,2pd)

Isomers	E_h_, Hartree	∆E, kcal/mol	μ_g_, D	*x*
1E	−1,574.7860481	0	10.5	0
1E′	−1,574.7860106	0.024	10.3	0
1Z	−1,574.7780105	5.04	7.52	0

**Table 3:** The S_0_ → S_1_ transition characteristics for the optimised isomers (Franck-Condon energy E_h_^FC^, transition energy ∆E, the oscillator strength f, the excited state dipole moment μ_e_, the state nature) calculated by TD-B3LYP/6–31 + G (3df,2pd)

Isomers	E_h_^FC^, Hartree	∆E_01_ (f), eV	μ_e_, D	State Nature
1E	−1,574.7317932	1.48 (0.04)	57.0	Charge Transfer
1E′	−1,574.7319603	1.47 (0.04)	56.1	Charge Transfer
1Z	−1,574.7240979	1.47 (0.02)	49.5	Charge Transfer

Both *trans*-isomers 1E and 1E′of DMPSDA possess a planar geometry of the TNP-amino fragment and a weak hybridized dimethylamino group on the opposite stilbene ring. TNP rings are non-planar to the stilbene plane of symmetry. Torsion angles of the TNP rings are 23.2° for 1E and 23.0° for 1E′. The crystal structure of 2,4,6-trinitro-N-[4-(phenyldiazenyl) phenyl] aniline shows the similar geometry of the TNP ring with the torsion angle of 27.7° [14].

**Fig. 5:**
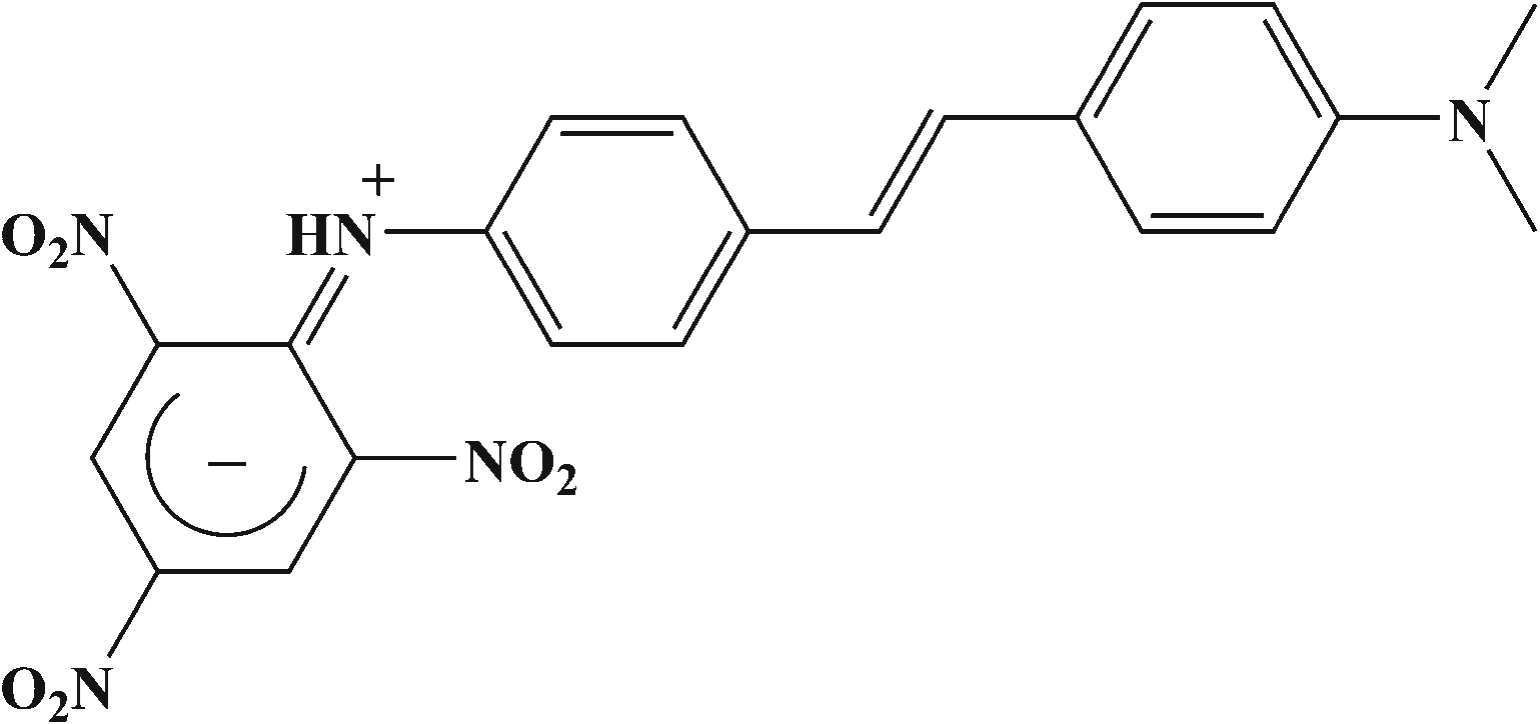
Betaine electronic structure of the TNP fragment of *trans*-DMPSDA

According to the calculations, the bond alternation of the *trans*-isomers is similar and slightly differs from the bond alternation of the *cis*-isomer 1Z, as seen in Fig. 4. The corresponding bond lengths of all calculated isomers are also similar. Bond distance between the nitrogen atom of the DMA group and the phenyl ring is 1.3789 Å (1E), 1.3790 Å (1E′) and 1.3816 Å (1Z). The bond lengths connecting the phenyl rings are 1.4595 Å, 1.3505 Å, 1.4561 Å (for 1E), 1.4594 Å, 1.3505 Å, 1.4561 Å (for 1E′) and 1.4703 Å, 1.3508 Å, 1.4654 Å (for 1Z). The bond alteration of the TNP ring in all isomers clearly demonstrates the betaine electronic structure of this fragment, shown in Fig. 5. According to the TD-B3LYP calculations for the optimized equilibrium structures, the long wavelength transitions S_0_ → S_1_ have a clear charge transfer nature. The dipole moments of the Franck-Condon states are listed in Table 3, which support the above conclusion.

## Conclusions

N,N-dimethyl-N′-picryl-4,4′-stilbenediamine (DMPSDA) was prepared in a form of lustrous black crystals, and its absorption and fluorescence spectra were recorded in cyclohexane, acetonitrile and dimethyl sulfoxide. Non-emissive intramolecular charge transfer (ICT) was clearly observed in this molecule at the longer wavelength in the absorption spectra in all three solvents. The measured fluorescence quantum yield values in all three solvents were found to be relatively low compared to the parent *trans*-4-dimethylamino-4′-aminostilbene. We assumed that part of the additional non-radiative losses are caused by the electronic energy transfer onto the TNP fragment, which intrinsically shows negligible fluorescence, due to the presence of an efficient conical intersection of the S_1_ state with the ground state S_0_. Theoretical calculations demonstrating a betaine electronic structure of the trinitrophenyl group in the ground state of the molecule and a charge transfer nature of the long wavelength transition S_0_ → S_1_ supported the aforementioned experimental observations of the ICT formation in the molecule.

## Abbreviations

DMPSDA: *N*,*N*-dimethyl-*N′*-picryl-4,4′-stilbenediamine
ICT: intramolecular charge transfer
DFT: density functional theory
LYP: correlation functional of Lee, Yang, and Parr
B3LYP: Becke’s three-parameter hybrid method with correlation functional of Lee, Yang, and Parr
TD-B3LYP: time-dependent B3LYP
DMA: dimethylamino
TNP: trinitrophenyl
EX: fluorescence excitation
EM: fluorescence emission
CH: cyclohexane
ACN: acetonitrile
DMSO: dimethyl sulfoxide
